# Neutrophilic dermatosis of the dorsal hands related to cocaine abuse^☆^^☆☆^

**DOI:** 10.1016/j.abd.2020.07.023

**Published:** 2021-07-13

**Authors:** Marcial Álvarez-Salafranca, Mar García-García, Begoña de Escalante Yangüela

**Affiliations:** a Department of Dermatology, Hospital Clínico Universitario Lozano Blesa, Zaragoza, Spain; b Department of Pathology, Hospital Clínico Universitario Lozano Blesa, Zaragoza, Spain; c Department of Internal Medicine, Hospital Clínico Universitario Lozano Blesa, Zaragoza, Spain

**Keywords:** Acute febrile, Cocaine-related disorders, Neutrophilic dermatosis, Prednisone, Sweet’s syndrome

## Abstract

Neutrophilic dermatoses encompass a wide spectrum of diseases characterized by a dense infiltration mainly composed of neutrophils. Neutrophilic dermatosis of the dorsal hands is currently considered a localized variant of Sweet syndrome. Cocaine abuse has been related to a wide range of mucocutaneous manifestations, including neutrophilic dermatoses such as pyoderma gangrenosum. The authors of this study present a patient with neutrophilic dermatosis of the dorsal hands, in which cocaine abuse was identified as a probable trigger.

## Introduction

Neutrophilic dermatoses are a group of heterogeneous cutaneous disorders characterized by dense epidermal, dermal, or hypodermal infiltration of neutrophils. Neutrophilic Dermatosis of the Dorsal Hands (NDDH), first described by Strutton et al., is considered a localized variant of Sweet Syndrome (SS). In the first description, the presence of leukocytoclastic vasculitis in skin biopsies led the authors to name it “pustular vasculitis of the hands”.^1^ However, current evidence suggests that vasculitis may represent an epiphenomenon similar to that observed in some cases of SS.^2^ Cocaine abuse has been related to a wide range of mucocutaneous manifestations.^3^ Here, the authors report a patient with a stereotypical clinical picture of NDDH, in which cocaine abuse seemed to be the trigger.

## Case report

A 46-year-old man with a previous history of type 1 diabetes mellitus, atrial fibrillation, dyslipidemia, and Graves-Basedow disease presented with a 5-days history of cutaneous lesions on the dorsal aspect of his hands, associated with thoracic pain, mild cough, and expectoration that had appeared simultaneously. The patient did not present fever nor other acute symptoms. He had not started taking new medications but recognized frequent use of cocaine for 8 months.

Chest radiography revealed right costophrenic angle blunting related to pleural effusion. In this sense, a diagnosis of atypical pneumonia was made and therefore treatment with amoxicillin-clavulanate and subsequently with azithromycin was indicated. Nevertheless, the patient showed no improvement. Thoracoabdominal Computed Tomography (TC) verified a diffuse bilateral infiltrate suggestive of small airway inflammatory disease and a discrete pleural effusion.

Serum biochemistry showed glycemic decompensation (299 mg/dL and HbA1C = 10.6%) and elevated acute phase reactants (Erythrocyte Sedimentation Rate – ESR = 58 mm; C-Reactive Protein – CRP = 4.55 mg/dL). Complete blood count revealed leukocytosis (14 mil/mm^3^) and neutrophilia (10.4 mil/mm^3^). Tumor markers and serum blood electrophoresis were within normal limits. Autoimmunity tests (including Antinuclear Antibodies [ANAs] and Extractable Nuclear antigen Antibodies [ENAs]), serological tests for human immunodeficiency virus, hepatitis B and C virus and respiratory tract infections (including *Chlamydia* *pneumoniae*, Legionella, *Mycoplasma* *pneumoniae*, Coxiella, and Respiratory Syncytial Virus) were negative.

**Figure 1:**
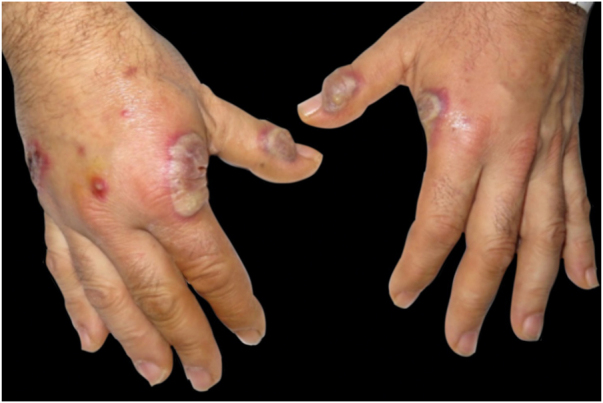
Clinical presentation. Eythematous-violaceous plaques, pustules, and bullous lesions on the dorsal hands.

**Figure 2:**
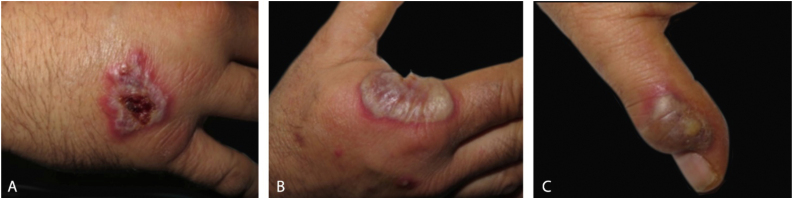
Detail of individual lesions of neutrophilic dermatosis of the dorsal hands (A), Crusted violaceous plaque (B), Large blister with an erythematous base on the radial aspect of the right hand (C), A lesion with purulent discharge over interphalangeal joint of the left hand.

**Figure 3:**
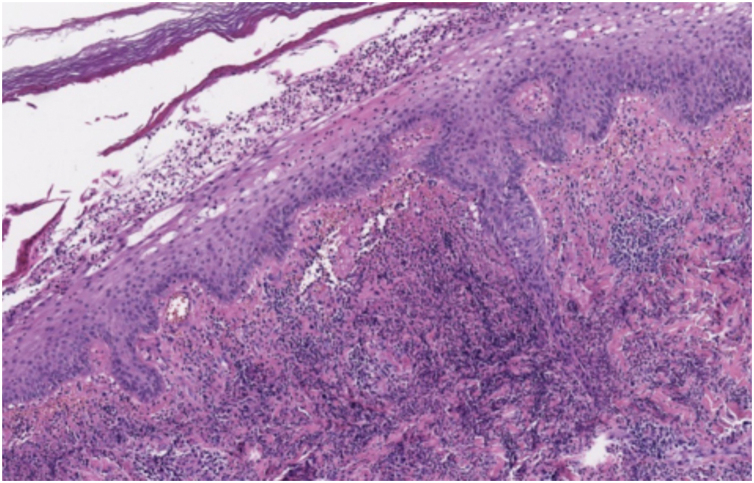
At low power, epidermis is acanthotic and there is subcorneal purulent material. A dense neutrophilic infiltrate is present in the upper dermis (Hematoxylin & eosin, ×8).

**Figure 4:**
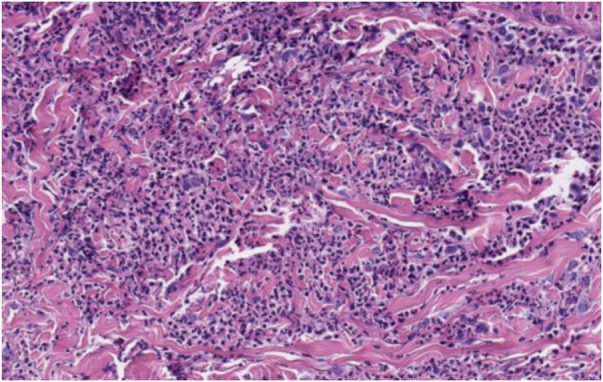
A high-magnification image showing a heavy dermal infiltrate of neutrophils (Hematoxylin & eosin, ×20).

Dermatological examination revealed bilateral erythematous-violaceous edematous plaques, partially ulcerated, and pustules coalescing in hemorrhagic bullous lesions on the dorsal aspect of his hands (Figure 1, Figure 2). Histopathological examination showed acanthosis with subcorneal neutrophilic blister formation and a dense dermal interstitial inflammatory infiltrate predominantly composed of neutrophils (Figure 3, Figure 4). In addition, karyorrhexis, erythrocyte extravasation, and subepidermal edema were present. A swab culture from skin bullae did not identify pathogenic microorganisms.

The patient was treated with oral prednisone 30 mg daily in a slow-tapering regimen for 8 weeks, along with topical betamethasone/gentamicin cream. Steroid therapy induced a dramatic response of both cutaneous lesions and thoracic pain. Moreover, the patient achieved to stop using cocaine since hospitalization to this day. During 1-year follow-up, no recurrence of the cutaneous lesions was observed, and the patient remained completely asymptomatic.

## Discussion

NDDH is currently considered an uncommon, localized variant of SS.^4^ Clinically, NDDH is characterized by erythematous-violaceous plaques and nodules, frequently ulcerated, predominantly involving the dorsal hands.^4^ This disorder has a predilection for the radial aspect of the hands and is generally bilateral.^5^ The localized distribution, clinical features and neutrophilic infiltration often lead to the initial diagnosis of cutaneous infection. However, the lesions do not respond to antibiotics and cultures are negative.^4^ Blood analysis usually reveals leukocytosis, neutrophilia and elevated ESR. Nevertheless, these findings are less common than in classical SS.^5^

Most cases of classic SS are idiopathic. Nevertheless, as in classical SS, there is a significant association between NDDH and systemic disease. In this sense, hematological disorders (mainly myelodysplastic syndrome and plasma cell dyscrasias), solid organ tumors, inflammatory bowel disease, and recent infections have been reported as triggers.^5^ For this reason, it is of paramount importance to perform a thorough physical examination and request complementary tests to rule out underlying systemic disease.

Pulmonary involvement in classic SS is rare. Skin lesions and pulmonary involvement are generally concomitant, being the most common symptoms of dyspnea and cough. Radiologic findings range from unilateral or bilateral interstitial infiltrates to lung opacities, with or without pleural effusion.^6^ When performed, cytological examination or biopsy can reveal a high proportion of neutrophils with negative cultures. In the present study’s case, these investigations were not performed because of the mild symptoms and its rapid improvement with steroids. To the authors’ best knowledge, there are no previous reports of suspected pulmonary involvement in NDDH.^5^

Histologically, NDDH is characterized by a dense dermal neutrophilic infiltrate with associated dermal edema.^5^ However, findings of leukocytoclastic vasculitis are more frequent than in classic SS. These findings are probably secondary to endothelial damage and are not necessary to make the diagnosis.^1^, ^5^, ^7^ Direct immunofluorescence is typically negative.

Although the spontaneous resolution has been rarely described, patients with NDDH usually receive active treatment. Although systemic corticosteroids are the mainstay of treatment, many patients benefit from the use of a steroid-sparing systemic agent in order to prevent recurrences, mainly dapsone. Other alternative drugs are colchicine, tetracycline or potassium iodide.^4^, ^5^

Mucocutaneous manifestations of cocaine abuse have been recently reviewed.^3^ Cocaine-induced vasculopathy is a well-described phenomenon that manifests as purpuric plaques predominantly involving the ears, nose and other facial regions. This purpuric eruption is related to microvascular thrombosis with or without leukocytoclastic vasculitis. Levamisole, a cocaine adulterant, has been identified as a trigger for the autoimmune response linked to this phenomenon.^3^ Regarding neutrophilic dermatoses, Pyoderma Gangrenosum (PG) secondary to chronic cocaine use is well documented and tends to present with multiple lesions.^8^, ^9^ Indeed, the strongest evidence for drug-induced PG concerns cocaine.^10^ The histologic examination may show vasculitic changes as a distinctive but no constant finding. It is not well known whether levamisole can be the pathogenic culprit behind cocaine-related PG.^8^, ^9^ Jeong et al. propose that levamisole is possibly implicated in the pathogenesis of cocaine-induced PG because of the similarity in the serologic profile of both entities. In this sense, p-ANCA (perinuclear antineutrophil cytoplasmic antibody) and antiphospholipid antibodies are frequently elevated in both vasculopathic levamisole toxicity and PG.^9^ Considering that neutrophilic dermatoses are frequently considered a continuum and there is a significant overlap between these disorders, it is reasonable to hypothesize that levamisole-adultered cocaine could be involved in the genesis of other neutrophilic cutaneous diseases.

In conclusion, the authors report a patient with NDDH, in which cocaine abuse was identified as a possible trigger. Cocaine use withdrawal along with appropriate acute phase treatment led to a prolonged time without recurrences and need for maintenance therapy. As far as the authors know, the relationship between cocaine and NDDH has not been previously reported.

## Financial support

None declared.

## Authors’ contributions

Marcial Álvarez-Salafranca: Approval of the final version of the manuscript; composition of the manuscript; collection, analysis, and interpretation of data; participation in the design of the study; critical review of the literature; critical review of the manuscript.

Mar García-García: Approval of the final version of the manuscript; composition of the manuscript; collection, analysis, and interpretation of data; participation in the design of the study; critical review of the literature; critical review of the manuscript.

Begoña de Escalante Yangüela: Approval of the final version of the manuscript; composition of the manuscript; collection, analysis, and interpretation of data; participation in the design of the study; critical review of the literature; critical review of the manuscript.

## Conflicts of interest

None declared.
